# Blood-brain barrier leakage hotspots collocating with brain lesions due to sporadic and monogenic small vessel disease

**DOI:** 10.1177/0271678X231173444

**Published:** 2023-05-03

**Authors:** Salvatore Rudilosso, Michael S Stringer, Michael Thrippleton, Francesca Chappell, Gordon W Blair, Daniela Jaime Garcia, Fergus Doubal, Iona Hamilton, Esther Janssen, Anna Kopczak, Michael Ingrisch, Danielle Kerkhofs, Walter H Backes, Julie Staals, Marco Duering, Martin Dichgans, Joanna M Wardlaw, on behalf of the SVDs@target consortium

**Affiliations:** 1 Comprehensive Stroke Center, Department of Neuroscience, Hospital Clinic and August Pi i Sunyer Biomedical Research Institute (IDIBAPS), Barcelona, Spain; 2 Centre for Clinical Brain Sciences, UK Dementia Research Institute, University of Edinburgh, Edinburgh, UK; 3 Department of Neurology, Radboud University Medical Centre (Radboudumc), Nijmegen, The Netherlands; 4 Institute for Stroke and Dementia Research, University Hospital, LMU Munich, Munich, Germany; 5 Department of Radiology, University Hospital, LMU Munich, Munich, Germany; 6 Department of Neurology and School for Cardiovascular Diseases (CARIM), Maastricht University Medical Center+, Maastricht, The Netherlands; 7 Department of Radiology & Nuclear Medicine, Schools for Mental Health & Neuroscience and School for Cardiovascular Diseases, Maastricht University Medical Centre, Maastricht, Netherlands; 8 Medical Image Analysis Center (MIAC AG) and Department of Biomedical Engineering, University of Basel, Basel, Switzerland; 9 Munich Cluster for Systems Neurology, Munich, Germany; 10 German Center for Neurodegenerative Diseases, Munich, Germany

**Keywords:** Blood-brain barrier, cerebral small vessel disease, dynamic-contrast enhanced imaging, lacunar, white matter hyperintensities

## Abstract

Blood-brain barrier (BBB) is known to be impaired in cerebral small vessel disease (SVD), and is measurable by dynamic-contrast enhancement (DCE)-MRI. In a cohort of 69 patients (42 sporadic, 27 monogenic SVD), who underwent 3T MRI, including DCE and cerebrovascular reactivity (CVR) sequences, we assessed the relationship of BBB-leakage hotspots to SVD lesions (lacunes, white matter hyperintensities (WMH), and microbleeds). We defined as hotspots the regions with permeability surface area product highest decile on DCE-derived maps within the white matter. We assessed factors associated with the presence and number of hotspots corresponding to SVD lesions in multivariable regression models adjusted for age, WMH volume, number of lacunes, and SVD type. We identified hotspots at lacune edges in 29/46 (63%) patients with lacunes, within WMH in 26/60 (43%) and at the WMH edges in 34/60 (57%) patients with WMH, and microbleed edges in 4/11 (36%) patients with microbleeds. In adjusted analysis, lower WMH-CVR was associated with presence and number of hotspots at lacune edges, and higher WMH volume with hotspots within WMH and at WMH edges, independently of the SVD type. In conclusion, SVD lesions frequently collocate with high BBB-leakage in patients with sporadic and monogenic forms of SVD.

## Introduction

Cerebral small vessel disease (SVD) is a disorder that affects perforating arterioles, capillaries, and venules in the brain that is present in more than half of the population aged 60 and almost 100% of subjects aged 90.^1^ Monogenic SVD variants like cerebral autosomal dominant arteriopathy with subcortical infarcts and leukoencephalopathy (CADASIL) and other less prevalent microangiopathies also affect younger populations.^2,3^ Although most perforating vessels are not directly visible using conventional neuroradiological imaging, typically brain tissue lesions that result from SVD are identifiable on magnetic resonance imaging (MRI), such as recent small subcortical infarcts, lacunes, white matter hyperintensities (WMH) of presumed vascular origin, perivascular spaces (PVS), and cerebral microbleeds (CMB).^4^

The SVD mechanisms are still not completely understood. However, more advanced imaging techniques, including increased magnetic field MRI (3 or 7 T), and techniques to assess microvascular function and blood-brain barrier (BBB) integrity, are yielding new insights into potential pathophysiological mechanisms in SVD.^5^ In particular, the study of BBB permeability using dynamic contrast-enhanced (DCE) MRI showed a link between BBB impairment and the severity of SVD.^6–9^ Tissue-type differences in BBB permeability have been described between gray and white matter, normal-appearing white matter (NAWM), WMH, and subcortical infarcts.^10,11^

**Figure 1.**
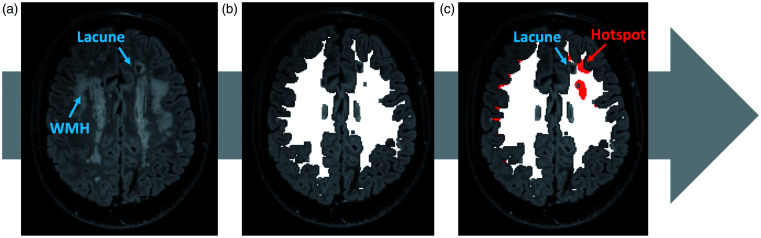
Imaging analysis of high permeability surface product hotspots corresponding to small vessel disease (SVD) features. (a) Visual assessment of SVD markers on structural MRI sequences; (b) White matter segmentation with peripheral 1 kernel voxel erosion (white mask) and (c) Identification of hotspots within the white matter segmentation spatially related to SVD feature, as indicated by the hotspots at lacune edges in the example. High permeability surface product hotspots represent regions with the highest PS decile value within the white matter segmentation.

Previous studies showed that focal spots of high BBB leakage may be identified within the white matter of patients with SVD, enabling a regional assessment of BBB integrity.^11,12^ Figure 1 shows how these spots appear on DCE-derived permeability surface area product (PS) maps and how they may be spatially related to tissue injury. Whether and to what extent hotspots of increased BBB leakage correspond to brain injury is still uncertain. We hypothesized that brain lesions due to SVD, such as lacunes, WMH, and microbleeds, correspond to areas of highest BBB leakage in the brain. In this study, we assessed whether regions with extremely high BBB leakage were associated with brain lesions characteristic of SVD, in patients with sporadic SVD and CADASIL.

## Materials and methods

### Study design and participants

This is a secondary analysis from a prospective multicentre observational cross-sectional study of patients with SVD enrolled in a study of neurovascular function measures assessed using standardized brain MRI called INVESTIGATE-SVD (Imaging NeuroVascular, Endothelial and STructural Integrity in prepAration to TrEat Small Vessel Diseases, ISRCTN 10514229). The study was approved by the local ethics committee in each participating institution and informed consent was obtained from all participants. The full protocol of the study is published.^13^ Data not provided in the article are available upon reasonable request.

The INVESTIGATE-SVD cohort included 45 patients with sporadic SVD recruited from centres in Edinburgh (UK, n = 25) and Maastricht (the Netherlands, n = 20), and 32 patients with CADASIL from a centre in Munich (Germany). In brief, the INVESTIGATE-SVD study included patients with symptomatic sporadic SVD (a lacunar ischaemic stroke in the last 5 years) or diagnosis of CADASIL. Patients with other causes of stroke, such as ≥50% carotid stenosis, cardioembolic source of embolism (i.e., atrial fibrillation), and other specific causes of stroke (i.e. haemorrhage, vasculitis, etc.) were not enrolled.^13^

For the current analysis, we selected patients with usable BBB permeability imaging (DCE-MRI), excluding patients with incomplete acquisitions or poor image quality (e.g. due to movement artefacts or contrast injection failure). We collected a full medical history, including demography, vascular risk factors (history of hypertension, diabetes mellitus, hyperlipidaemia, history of ischaemic heart disease and peripheral vascular disease, current smoking habit, alcohol intake), and current prescribed medications.

### Image acquisition

Images were acquired using 3T MRI scanners. The total time to acquire all MRI sequences was approximately 1 hour and 45 minutes, including T1-weighted, T2-weighted, FLuid-Attenuated Inversion Recovery (FLAIR), and Susceptibility-Weighted Imaging (SWI) structural sequences. Imaging parameters are described elsewhere.^13^

BBB permeability was assessed using T1-weighted Dynamic Contrast-Enhanced (DCE) MRI. Patients received an intravenous bolus injection of Gadobutrol (Gadovist, Bayer, Germany), at a dose of 0.1 mmol/kg. BBB leakage was measured as permeability surface area product (PS) using the Patlak model.^13,14^

Cerebrovascular reactivity was assessed using Blood-Oxygen-Level-Dependent (BOLD) MRI with CO_2_ challenge. A single-shot GE echo planar imaging sequence with whole brain coverage was used to acquire images during a 12-minute breathing paradigm (alternating 2 minutes of air and 3 minutes of 6% CO_2_).^13^

### Image analysis

An analyst (EJ) not involved in the clinical assessment identified the radiologic markers of SVD, that is, lacunes, PVS, CMB, and WMH, on structural MRI sequences according to STRIVE criteria^4^ and graded them using validated qualitative scales for WMH (Fazekas scale)^15^ and PVS load scale in basal ganglia and centrum semiovale.^16^ Total SVD score was calculated on an ordinal scale from 0 to 4, by counting the presence (one point) of one or more lacunes, one or more CMB, confluent deep WMH (Fazekas score 2 or 3) or periventricular WMH extending into the deep white matter (Fazekas score 3), moderate to severe grade (2–4) of perivascular spaces in basal ganglia.^17^

Normal appearing white matter (NAWM) and WMH were segmented using a semi-automated pipeline.^18^ We obtained intracranial, NAWM, and WMH volumes. We normalized WMH volume to intracranial volume (ICV; WMH volume/intracranial volume) to limit inter-individual variability due to age and sex. Each vascular function measure, including PS, plasma volume (vP), and CVR magnitude, was obtained from NAWM and WMH regions.^13^ The vascular input function was obtained from the superior sagittal sinus.

The PS analysis was restricted to the white matter excluding the brainstem, applying one voxel-kernel erosion in native space to the white matter segmentation, as previously described.^19^ The cortical grey matter was excluded from the analysis because 2-mm spatial resolution is insufficient to avoid substantial partial volume artefact from white matter, CSF, and superficial vessels. In a random sample of 5 patients, we tested several combinations of PS thresholds (highest 5, 10, 15, and 20 percentile) to identify the areas with high PS and a minimum number of voxels (2, 5, and 10). We aimed to determine an optimal threshold that avoided very small spots with a difficult interpretation that might correspond to artefacts or background noise. Very restrictive PS thresholds (5 percentile) produced maps of difficult interpretations because areas with high PS values appeared fragmented in many non-confluent areas. Less restrictive percentiles such as 15 and 20 produced very large implausible confluent areas. Therefore, regions with the highest 10% PS values (highest decile) and a minimum threshold of 5 contiguous voxels were defined as hotspots. We preferred to use relative PS thresholds rather than absolute values due to the interindividual variability in PS values.

The PS maps and other structural sequences were co-registered to the T2-w MRI scan using FSL-FLIRT to enable the visual assessment of the tissue features corresponding to the hotspots (Figure 1).^20^

The number of hotspots was obtained automatically from the segmentation.

A stroke neurologist (SR) evaluated the maps showing the hotspots and described their distribution and load (absence, low, medium, high; details on Supplemental Material) according to anatomical brain regions: cerebellum, internal capsule, temporal lobes, white matter areas anterior and posterior to corresponding anterior and posterior lateral ventricle horns and centrum semiovale comprised between anterior and posterior horns.

The hotspots corresponding to SVD features (hotspots at lacune edges, hotspots within WMH, hotspots at WMH edges, and hotspots at CMB edges) were identified and counted on PS maps using a 3D image viewer (FSLeyes^©^ Copyright 2022, Paul McCarthy, University of Oxford, Oxford, UK) to overlay segmentations on structural sequences. These hotspots spatially closely related to SVD features were evaluated in specific subsets of patients presenting the corresponding SVD lesion in the white matter on the more appropriate MRI sequence according to STRIVE criteria (lacunes and WMH on FLAIR, and CMB on SWI sequences), as shown in Figure 1. Therefore, a hotspot could be identified at a lacune edge and be within a WMH at a time, and be included in both lacune and WMH analysis subgroups. For the analysis of hotspots related to lacunes, we excluded patients without lacunes in the segmented white matter. We defined as hotspots at lacune edges wherever a hotspot in the white matter was boarding, completely or partially, a lacune (which was not segmented as filled by CSF) or was at least in direct contact with part of the lacune board. For the analysis of hotspots related to WMH, we selected patients with the presence of an evaluable load of both WMH and NAWM, including patients with more than minimal WMH load (deep WMH Fazekas score > 0 or periventricular WMH Fazekas score > 1), and excluding patients with large confluent WMH with minimal NAWM left, because, if included, almost none or all hotspots would be located within WMH, respectively. For the analysis of hotspots at CMB edges we excluded patients without CMB in the segmented white matter and identified them using the same criteria for hotspots at lacune edges. To improve the accuracy of the visual assessment, this evaluation was performed twice by the same reader at different times. The agreement for hotspots at lacune edges was almost perfect (linearly weighted kappa = 0.95), perfect for hotspots at CMB edges (100% agreement), while was substantial for hotspots within WMH (linearly weighted kappa = 0.71) and hotspots at WMH edges (linearly weighted kappa = 0.68).

### Statistical analysis

Quantitative continuous variables with normal distribution are presented as mean and standard deviation (SD). Continuous variables without normal distribution and ordinal variables are presented as median and interquartile range (IQR). Binary variables are presented as absolute numbers and relative frequency (%). Means and medians between groups were compared using Student t, ANOVA, or Mann-Whitney U tests, as appropriate. Categorical variables were compared using Pearson’s chi-square or Fisher’s exact test as appropriate, and ordinal variables using Spearman correlation. Continuous variables without normal distribution (normalized WMH volume) were log 10 transformed in the multivariable analysis. No missing values were detected among clinical and structural imaging variables. CVR data were lacking in 6 patients in the whole cohort of selected patients for the main analysis (4 in lacune, 5 in WMH, and 0 in CMB subgroups).

We assessed the demographic, clinical, and imaging factors in univariable analysis according to the presence of one or more hotspots spatially corresponding to each SVD (lacunes, WMH and CMB). Then, we assessed the associations of the variables with p < 0.1 in univariable analysis and vascular function measures (PS, vP, CVR) in per-patient multivariable logistic and ordinal (number) multivariable regression analysis for the presence and number of hotspots corresponding to each SVD marker. The multivariable analyses were adjusted for pre-established covariates in model 1 (age, log10 normalized WMH volume, number of lacunes, CMB) and model 2 (model 1 + SVD type). We checked the collinearity of the variables introduced in the multivariable models, considering acceptable a Variance Inflation Factor <5.^21^ We considered as statistically significant p < 0.05, and all hypotheses were two-sided. All statistical analyses were performed using Stata/IC 15.1 for Mac (StataCorp, College Station, TX).

## Results

**Figure 2.**
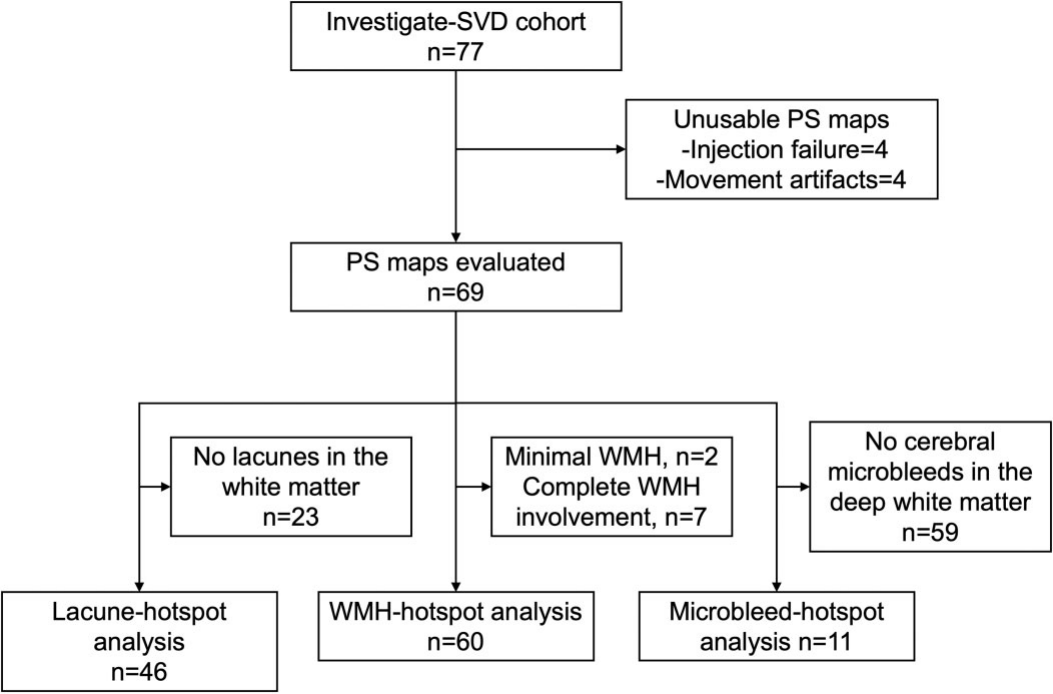
Flow diagram presenting the selection process for the analysis. PS: permeability surface product; WMH: white matter hyperintensities.

Amongst the 77 patients of the INVESTIGATE-SVD cohort, 69 patients had usable PS maps (Figure 2). Compared to those with sporadic SVD, patients with CADASIL were younger, less likely to have hypertension and hyperlipidaemia, and less likely to receive antiplatelet therapy. Patients with CADASIL had a higher load of structural SVD markers (Table 1).

**Table 1.** Main clinical and radiological characteristics of the study cohort and according to the subtype of small vessel disease.

	Whole cohort, N = 69	Sporadic SVD, N = 42	CADASIL, N = 27	P value
Age, y, mean (SD)	59.6 (12.5)	63.9 (10.7)	52.9 (11.4)	<0.001
Female, n (%)	31 (44.9)	18 (42.9)	13 (48.2)	0.666
Ischaemic heart disease, n (%)	8 (11.6)	7 (16.7)	1 (3.7)	0.136
History of Diabetes Mellitus, n (%)	10 (14.5)	9 (21.4)	1 (3.7)	0.076
History of hypertension, n (%)	44 (63.7)	34 (81.0)	10 (37.0)	<0.001
History of hyperlipidaemia, n (%)	41 (59.4)	31 (73.8)	10 (37.0)	0.002
Alcohol use, n (%)	49 (71.0)	29 (69.1)	20 (74.1)	0.653
Current smoking habit, n (%)	35 (50.7)	21 (50.0)	14 (51.9)	0.881
Antiplatelet therapy, n (%)	56 (81.2)	40 (95.2)	16 (59.3)	<0.001
Lacunes, median (IQR)	3 (0–6)	1 (0–4)	5 (1–9)	0.006
PVH Fazekas score, median (IQR)	2 (1–3)	2 (1–2)	3 (3–3)	<0.001
DWMH Fazekas score, median (IQR)	2 (1–3)	1 (1–2)	3 (2–3)	<0.001
PVS in basal ganglia, median (IQR)	2 (1–3)	2 (1–3)	3 (2–4)	0.002
PVS in centrum semiovale, median (IQR)	3 (2–3)	2 (1–3)	3 (2–4)	0.001
CMB, median (IQR)	0 (0–3)	0 (0–2)	1 (0–7)	0.037
Total SVD score, median (IQR)	3 (2–4)	3 (1–3)	4 (3–4)	0.002
WMH volume, mL, median (IQR)	13.2 (5.7–58.2)	8.2 (4.0–12.8)	76.5 (43.7–147.4)	<0.001
PS mean NAWM * 10^4^, mean (SD)	0.43 (1.14)	0.93 (0.94)	−0.33 (0.99)	<0.001
PS mean WMH * 10^4^, mean (SD)	0.91 (1.11)	1.09 (1.20)	0.65 (1.11)	0.120
vP mean NAWM * 10^4^, mean (SD)	41.42 (14.69)	47.06 (13.98)	32.74 (11.26)	<0.001
vP mean WMH * 10^4^, mean (SD)	62.30 (27.63)	72.04 (23.46)	47.33 (27.23)	<0.001
CVR mean NAWM * 10^2^, mean (SD)	3.57 (3.34)	3.59 (3.19)	3.55 (3.58)	0.958
CVR mean WMH * 10^2^, mean (SD)	5.83 (6.59)	7.52 (6.25)	3.64 (6.47)	0.020

SVD: small vessel disease; CADASIL: cerebral autosomal dominant arteriopathy with subcortical infarcts and leukoencephalopathy; NAWM: normal appearing white matter; WMH: white matter hyperintensities; PVH: periventricular hyperintensities; DWHM: deep white matter hyperintensities; PVS: perivascular spaces; CMB: cerebral microbleeds; Normalized WMH: WMH volume/intracerebral volume; PS: permeability surface product; vP: vascular plasma volume; CVR: cerebrovascular reactivity. CVR measures were calculated among the 60 patients with full data available.

We identified 3 subgroups of patients according to the presence of: a) at least one lacune in the deep white matter (N = 46, 22 (48%) patients with CADASIL); b) evaluable WMH (N = 60, 20 (33%) patients with CADASIL) excluding those with minimal WMH (N = 2) and large confluent WMH with minimal NAWM left (N = 7); and c) at least one microbleed in the deep white matter (N = 10, 5 (50%) patients with CADASIL), as represented in (Figure 2).

Compared to patients with sporadic SVD, patients with CADASIL had lower mean PS in the NAWM, but not in WMH, lower mean vP in both NAWM and WMH, and lower mean CVR magnitude in the WMH but not in NAWM (Table 1).

**Figure 3.**
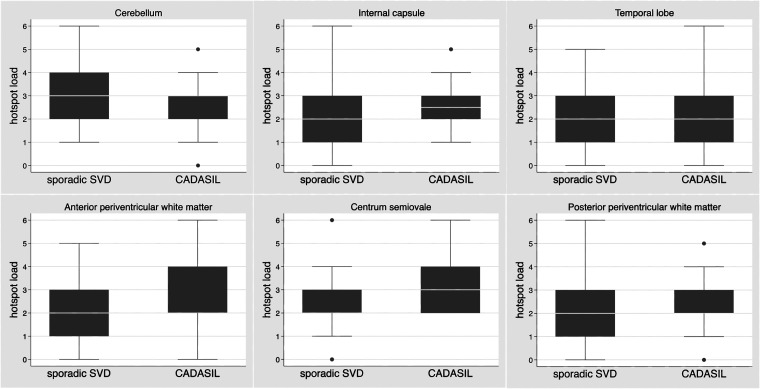
Distribution of the hotspots among the prespecified regions of the brain according to sporadic SVD and CADASIL type. Boxplot of the semiquantitative scale of hotspots for each brain region (sum of left and right side, range 0 to 6). Only for centrum semiovale region the hotspots load was higher in patients with CADASIL compared to sporadic SVD (p = 0.004).

Overall, the per-patient mean (SD) total number of hotspots was 69.1 (26.6), which was higher in patients with CADASIL [86.0 (29.7)] compared with sporadic SVD [58.2 (17.6)], p < 0.001, and in patients with a higher grade of total SVD score (Spearman Rho = 0.297, p = 0.013). The distribution of the hotspots among the prespecified regions of the brain was similar between sporadic SVD and CADASIL, except for centrum semiovale where patients with CADASIL had more hotspots than patients with sporadic SVD (p = 0.004), as shown in Figure 3.

### BBB leakage hotspots corresponding to lacunes in deep white matter

**Figure 4.**
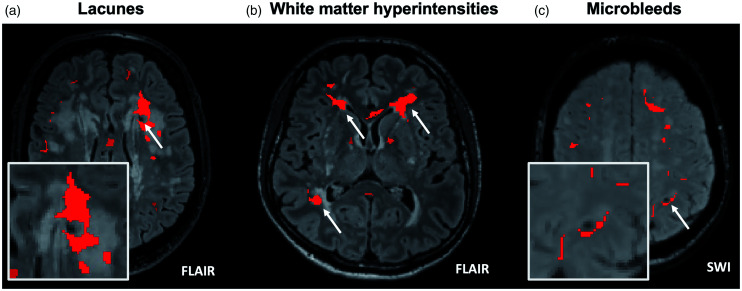
Representative images of hotspots corresponding to each SVD feature. (a) A lacune in the left anterior periventricular region is surrounded by a hotspot. (b) WMH areas with hotspots within or at the edges and (c) A lobar cerebral microbleed in the left parieto-frontal lobe is at edges of a hotspot.

**Figure 5.**
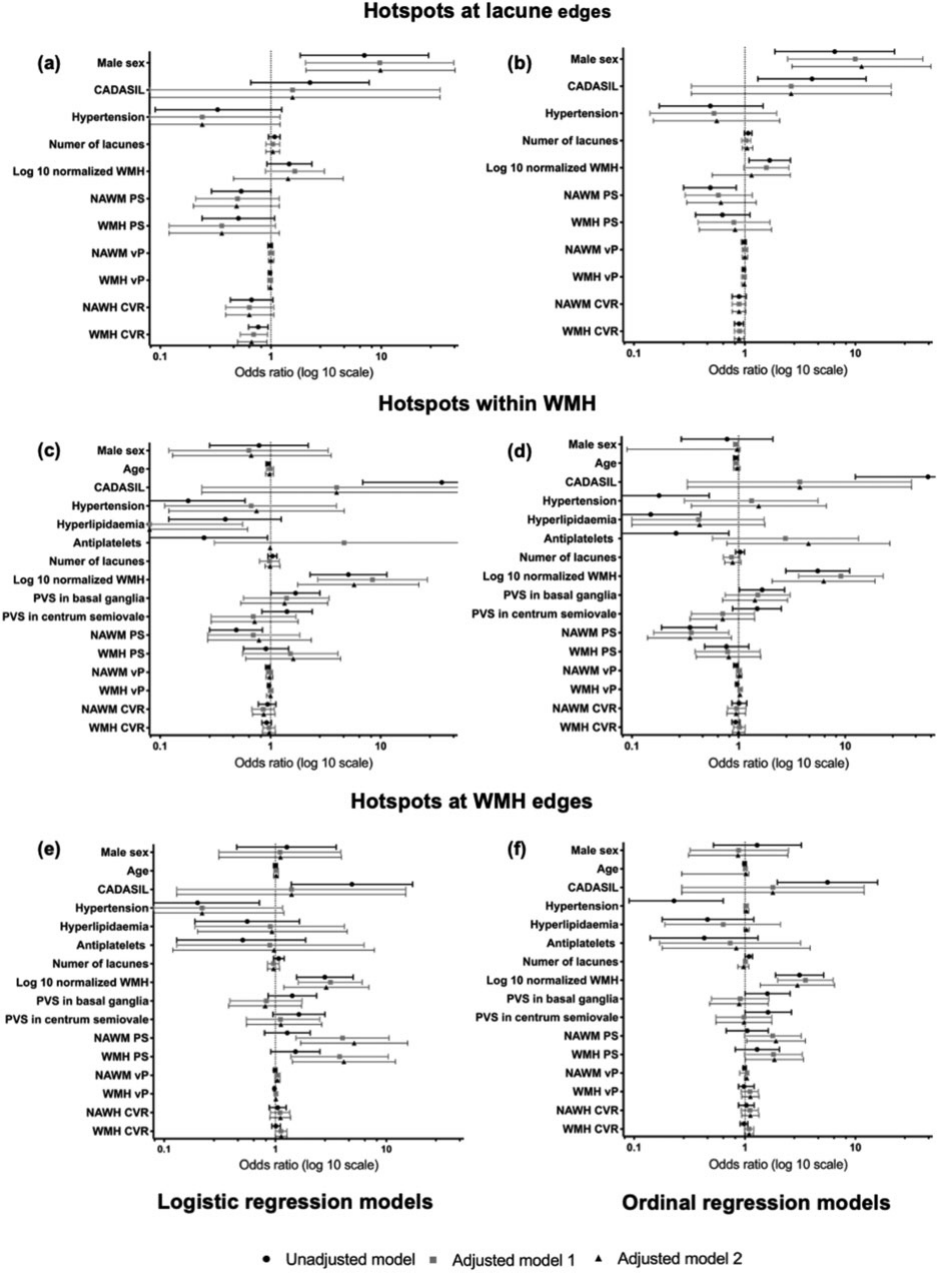
Forest plots summarizing the regression analyses of the factors associated with the presence (logistic regression) and number (ordinal regression) of hotspots related to lacunes and WMH. CADASIL: cerebral autosomal dominant arteriopathy with subcortical infarcts and leukoencephalopathy; WMH: white matter hyperintensities; NAWM: normal appearing white matter; PVS: perivascular spaces; PS: permeability surface product; vP: vascular plasma volume; CVR: cerebrovascular reactivity; model 1: age, log10 normalized WMH volume, number of lacunes, cerebral microbleeds; model 2: (model 1 + SVD type).

Amongst the subgroup of 46 patients (22 patients with CADASIL and 24 patients with sporadic SVD) having at least one lacune in deep white matter (median 2, IQR 1-6)) 29/46 patients (63.0%) had at least one lacune with a hotspot at its edges (median 1, IQR 1-3). Figure 4(a) shows a representative image of a lacune corresponding to a hotspot. In this subgroup analysis, patients with CADASIL were younger, were less likely to have history of hypertension and hyperlipidaemia, and be on antiplatelet therapy, had more severe SVD (lacunes, WMH and PVS), had overall lower PS, vP, and CVR values, and more hotspots compared to patients with sporadic SVD (Supplemental Table 1). Among the 194 lacunes in the deep white matter evaluated, 59 (30.4%) corresponded to a hotspot. In univariable analysis, patients with hotspots at lacune edges were more frequently male, had lower PS in NAWM, and lower CVR in both NAWM and WMH (Table 2). No relevant collinearity was detected among the variables introduced in the multivariable model 1 (highest VIF = 1.20) and 2 (highest VIF = 4.09, mainly due to collinearity between WMH volume and type of SVD). In the multivariable logistic and ordinal regression models 1 and 2 (Figure 5(a) and (b); Supplement Table 2, and 3), male sex and lower CVR in WMH were the only variables that showed a significant association with the presence and number of hotspots at lacune edges, respectively.

**Table 2.** Univariable clinical and radiological characteristics of the study cohort and according to the presence of hotspots associated with SVD features in different subgroups.

	No hotspots at lacune edges, N = 17 (37%)	≥1 hotspots at lacune edges, N = 29 (63%)	p	No hotspots within WMH, N = 34 (57%)	≥1 hotspots within WMH, N = 26 (43%)	p	No hotspots at WMH edges, N = 26 (43%)	≥1 hotspots at WMH edges, N = 34 (57%)	p
CADASIL, n (%)	6 (35.3)	16 (55.2)	0.193	2 (5.9)	18 (69.2)	<0.001	4 (15.4)	16 (47.1)	0.010
Age, y, mean (SD)	59.5 (9.29)	58.5 (10.7)	0.759	62.0 (11.1)	55.0 (13.9)	0.033	59.0 (11.0)	59.0 (14.1)	0.998
Female, n (%)	11 (64.7)	6 (20.7)	0.003	15 (44.1)	13 (50.0)	0.651	13 (50.0)	15 (44.1)	0.651
Ischaemic heart disease, n (%)	2 (11.8)	3 (10.3)	1.000	5(14.7)	3 (11.5)	0.721	3 (11.5)	5 (14.7)	0.721
Diabetes, n (%)	3 (17.7)	1 (3.5)	0.135	8 (23.5)	2 (7.7)	0.103	6 (23.1)	4 (11.8)	0.305
Hypertension, n (%)	13 (76.5)	15 (51.7)	0.097	28 (82.4)	12 (46.2)	0.003	22 (84.6)	18 (52.9)	0.010
Hyperlipidaemia, n (%)	12 (70.6)	15 (51.7)	0.210	28 (82.4)	9 (36.6)	<0.001	18 (69.2)	19 (55.9)	0.292
Alcohol use, n (%)	13 (76.5)	22 (75.9)	1.000	23 (67.7)	18 (69.2)	0.896	16 (61.5)	25 (73.5)	0.322
Smoker, n (%)	8 (47.1)	16 (55.2)	0.595	16 (47.1)	12 (46.2)	0.994	11 (42.3)	17 (50.0)	0.554
Antiplatelet, n (%)	12 (70.6)	23 (79.3)	0.722	30 (88.2)	17 (65.4)	0.033	22 (84.6)	25 (73.5)	0.302
Lacunes total, median (IQR)	3 (1–5)	6 (3–9)	0.087	1 (0–3)	4 (1–8)	0.026	1 (0–4)	3 (1–7)	0.074
PVH Fazekas score, median (IQR)	2 (1–3)	3 (2–3)	0.021	1.5 (1–2)	3 (2–3)	<0.001	2 (1–2)	3 (2–3)	<0.001
DWMH Fazekas score, median (IQR)	2 (1–2)	3 (2–3)	0.046	1 (1–2)	2.5 (2–3)	<0.001	1 (1–2)	2 (2–3)	<0.001
PVS in basal ganglia, median (IQR)	3 (2–3)	3 (2–4)	0.981	2 (1–3)	3 (2–3)	0.038	1.5 (1–2)	2 (1–3)	0.109
PVS in centrum semiovale, median (IQR)	3 (2–3)	3 (2–4)	0.220	2 (1–3)	2.5 (2–3)	0.188	2 (1–3)	3 (2–3)	0.080
CMB, median (IQR)	1 (0–5)	2 (0–7)	0.188	0 (0–2)	1 (0–3)	0.277	0 (0–2)	0.5 (0–3)	0.507
WMH volume, mL, median (IQR)	12.8 (5.1–43.7)	46.7 (14.6–105.83)	0.057	8.18 (4.01–11.97)	40.13 (18.76–73.76)	<0.001	6.20 (3.75–11.41)	27.80 (11.68–65.95)	<0.001
PS mean NAWM * 10^4^, mean (SD)	0.90 (0.99)	0.19 (1.15)	0.043	0.81 (1.02)	0.07 (1.15)	0.007	0.36 (1.20)	0.68 (1.10)	0.301
PS mean WMH * 10^4^, mean (SD)	1.32 (1.04)	0.76 (0.90)	0.067	1.04 (1.26)	0.92 (0.95)	0.706	0.71 (1.43)	1.21 (0.79)	0.099
vP mean NAWM * 10^4^, mean (SD)	40.40 (8.67)	37.98 (16.48)	0.581	47.02 (14.42)	37.25 (12.72)	0.010	43.55 (14.60)	42.40 (14.55)	0.770
vP mean WMH * 10^4^, mean (SD)	61.42 (18.28)	50.72 (25.03)	0.135	73.45 (28.03)	54.50 (21.44)	0.008	74.04 (29.18)	58.77 (23.41)	0.033
CVR mean NAWM * 10^2^, mean (SD)	4.95 (1.66)	2.45 (4.42)	0.042	3.96 (1.42)	3.32 (4.59)	0.468	3.40 (3.81)	3.90 (2.62)	0.569
CVR mean WMH * 10^2^, mean (SD)	8.97 (4.25)	2.62 (7.00)	0.003	7.62 (5.21)	4.74 (7.30)	0.097	6.09 (6.13)	6.62 (6.49)	0.765
Number of hotspots, median (IQR)	57 (54–95)	65 (57–87)	0.274	58 (48–69)	64.5 (50–83)	0.244	57 (47–72)	61 (51–86)	0.205

CADASIL: cerebral autosomal dominant arteriopathy with subcortical infarcts and leukoencephalopathy; NAWM: normal appearing white matter; WMH: white matter hyperintensities; PVH: periventricular hyperintensities, DWHM: deep white matter hyperintensities; PVS: perivascular spaces; CMB: cerebral microbleeds; Normalized WMH: WMH volume/intracerebral volume; PS: permeability surface product; vP: vascular plasma volume; CVR: cerebrovascular reactivity, hotspots: PS spots according to highest 10% PS values in segmented WM. CVR measures were calculated among the 60 patients with full data available.

### BBB leakage hotspots related to WMH

This analysis was performed in 60 patients (20 patients with CADASIL and 40 patients with sporadic SVD), after the exclusion of 7 patients with almost complete WMH involvement of the white matter leaving no NAWM for comparison with WMH (all of them with CADASIL), and 2 patients with very minimal WMH. In this subgroup analysis, patients with CADASIL were younger, were less likely to have history of hypertension and hyperlipidaemia, and be on antiplatelet therapy, had more severe SVD (WMH and PVS), had overall lower PS, vP values, and more hotspots compared to patients with sporadic SVD (Supplemental Table 1). We identified 26/60 patients (42.6%) with one or more hotspots within WMH and 34/60 patients (56.7%) with one or more hotspots at WMH edges (Figure 4(b)). In the univariable analysis (Table 2), among clinical variables, CADASIL SVD type, and absence of history of hypertension, were associated with both hotspots within and at WMH edges, while, younger age, absence of history of hyperlipidaemia and of antiplatelet use were associated with hotspots within WMH but not with hotspots at WMH edges. Among imaging variables, qualitative and quantitative variables describing WMH volume, and lower mean vP in WMH were associated with hotspots located both within and at WMH edges, while number of lacunes, PVS in basal ganglia, lower mean PS in NAWM and lower mean vP in NAWM were associated with hotspots within WMH but not with those at WMH edges (Table 2).

No relevant multicollinearity was found among co-variates in the multivariable models (highest VIF in model = 1.29 and in model 2 = 3.37). In the multivariable regression analyses (Figure 5(c) and (d), Supplement Tables 4 and 5) for hotspots within WMH, WMH volume was associated with the presence and number of hotspots in all regression models. The absence of hyperlipidaemia was significant in logistic regression models 1 and 2, but not in ordinal regression models. Younger age was significant only in model 1 logistic regression but was no longer significant after adding SVD type to the model (model 2). Lower mean PS values in NAWM was significant in ordinal regression models 1 and 2.

In the multivariable logistic and ordinal regression analyses for hotspots at WMH edges (Figure 5(e) and (f), Supplement Tables 6 and 7), WMH volume had a strong positive association in all models; higher mean PS values in NAWM and WMH had a significant association in all models, except for ordinal regression model 1 (marginally-significant positive trend).

### BBB leakage spots related to cerebral microbleeds

Eleven patients, 6 (55%) with CADASIL and 5 (45%) with sporadic SVD, presented CMBs within the segmented white matter (4 patients with 1 CMB, 1 patient with 2 CMBs, 4 patients with 3 CMBs, and 2 patients with 4 CMBs). Patients with CADASIL had larger WMH volume, more PVS, and lower vP values compared to patients with sporadic SVD (data not shown). Four patients (36%), 2 with CADASIL and 2 with sporadic SVD, presented at least one CMB associated with a hotspot (Figure 4(c)). Overall, 5/26 (19%) of the CMBs in the white matter corresponded to a hotspot. The small number of patients precluded the possibility of exploring associations in multivariable analysis.

## Discussion

In this study, we analyzed the characteristics of the regions with the highest BBB permeability in patients with sporadic or monogenic SVD and their spatial relationship with lacunes, WMH, and microbleeds. Patients with CADASIL had more hotspots compared to patients with sporadic SVD, but this association was due to the severity of SVD rather than the type of SVD. Focal lesions such as lacunes and CMB showed hotspots in more than half of cases, and about half of patients with WMH showed hotspots within or at WMH edges. The presence and number of hotspots co-located with lacunes were related to male sex and lower CVR in WMH, while the presence and number of hotspots at WMH edges were related to overall higher PS values in NAWM and WMH.

The relationship between SVD severity and altered BBB permeability, usually in large tissue-defined regions of interest, has been previously described in several studies using DCE-MRI.^6–9^ This study adds new information on the pattern of BBB alterations in relation to brain lesions due to SVD, and in relation to alterations in CVR, in patients with sporadic and monogenic forms of SVD.

The number of hotspots was higher in patients with CADASIL compared with sporadic SVD, especially in centrum semiovale. However, patients with CADASIL had higher SVD lesion volumes and larger surface areas, and the association between SVD type and hotspot number was lost after adjusting for age, sex, and load of SVD markers. This suggests that the presence of spots of highly impaired BBB is a function of the overall SVD severity and stage rather than different pathophysiological mechanisms due to specific SVD aetiology. For this reason, and given the small sample size of each subgroup and the collinearity of the type of SVD with SVD markers, the effect of the SVD type in each subgroup analysis might be difficult to interpret. Therefore, we decided to include in each multivariable model the most relevant SVD markers, such as the number of lacunes, WMH volume and microbleeds, in addition to age, while the type of SVD was added only to model 2.

About two-thirds of patients with lacunes in the white matter had hotspots at the edges of the cavitation, which globally occurred in 30% of the lacunes in the white matter. However, PS values in the white matter around lacunes not corresponding to a hotspot have not been assessed in this study and BBB permeability might be increased without reaching the PS threshold adopted to define hotspots. We do not know the age of the lacunes in this cross-sectional study; longitudinal studies should assess BBB leakage in relation to lesion formation. This finding was associated with global lower CVR, especially in the WMH. This result is consistent with the results of a recent study in the same cohort of patients showing altered CVR in the white matter surrounding cavities that show clusters thought to be dilated small vessels on SWI, which seems to presage tissue destruction and lacune formation.^22^

Male sex was associated with hotspots corresponding to lacunes but not with WMH in multivariable models in both logistic and ordinal regressions. In this cohort, there was a strong association of male sex with the presence of hotspots corresponding to any SVD markers in the univariable analysis, which could be in part due to the higher number of lacunes in males compared to females. Epidemiological data indicate that severe SVD is more common in males than females.^23^ A large metabolome study in more than 9000 individuals from different populations showed differences in lipid metabolites associations with WMH in men versus women, potentially accounting for this male:female difference and suggesting that lipid differences according to sex could have a role in BBB integrity,^24^ potentially accounting for the male:female difference in severe SVD. Nevertheless, the influence of sex on BBB integrity and associations with different features of SVD are unexplored and warrant further investigation.^23^

The presence of vascular risk factors, such as history of hypertension and hyperlipidaemia, and the likelihood of being on antiplatelet therapy showed univariable associations with SVD-related hotspots that were no longer significant after adjusting for covariates, except for history of hyperlipidaemia in multivariable logistic analysis for hotspots within WMH. These findings are probably due to different vascular risk factors profiles between patients with sporadic and monogenic SVD.

Overall, hotspots in the white matter seem to be randomly distributed within subcortical white matter, and around half of them appeared to be located within WMH or surrounding them, as previously described.^11,12^ As expected, the presence and number of these hotspots spatially related to WMH depended on the load of WMH and globally higher PS values, but not with other SVD markers or vascular function measures (an association with lacunes reduced after co-variate adjustment). This may reflect that WMH are the dominant SVD lesion, with other lesions being less frequent. Since WMH severity is a main risk factor for WMH worsening over time, an association of WMH with PS is consistent with a major role for BBB dysfunction in SVD progression in both sporadic and monogenic SVD. Longitudinal studies analysing the fate of the white matter tissue at WMH edges in relation with regional PS values might shed light on the role of BBB impairment in incident lacune formation on WMH edges previously described in patients with CADASIL.^25^

This study showed that highly altered BBB may also correspond to CMB, in both patients with sporadic and monogenic types of SVD, consistent with recent post-mortem analysis of CMB^26^ and with previous works demonstrating high levels of Matrix Metalloproteinases in cerebrospinal fluid, suggesting altered BBB in patients with CAA and CMB.^27^ The presence of high PS values around a CMB, as well as for other SVD lesions, might also be related to the timing of the CMB appearance that could not be assessed in this study. However, the low prevalence of CMB in the white matter in this cohort hampers the interpretation of these findings. Further studies assessing imaging markers of BBB impairment in patients with a high load of CMB (i.e. CAA) are needed.

To assess the BBB function in patients with SVD we used the Patlak model, which is based on the assumptions of high cerebral blood flow relative to the BBB leakage rate and negligible backflux of the contrast agent. Previous work demonstrated the robustness and validity of the Patlack model approach for the assessment of BBB impairment in SVD.^28^ However, it is to be determined whether pathophysiological differences between sporadic SVD and CADASIL, i.e. different arteriolar thickness and blood pressure pulsatility, might affect the applicability of the Patlak model assumption.

The main strengths of this study are the homogeneity of the image protocols used in three different centers. The image analysis is based on validated acquisition and processing protocols according to HARNESS initiative recommendations,^14,29^ the image analysis to derive WMH and NAWM, visual assessments and hotspot detection were independent of each other and blind to clinical variables. Careful visual image analysis for the identification of the hotspots was done after several objective tests to select the best PS thresholds with an optimal balance of sensitivity and specificity. In addition, the rigorous assessment of patients with well-defined sporadic and monogenic SVD who were included allowed assessment and some differentiation of the impact of SVD type and severity according to the main findings on imaging.

However, there are several limitations that may be relevant for the interpretation of the results. First, the evaluation of the hotspots according to the different SVD markers depended on one observer. However, intra-observer reliability was acceptable, independent of PS map preparation, and blinded to other clinical and radiological variables. In addition, representative cases were discussed with an expert neuroradiologist (JMW). Second, selecting the last decile PS value cutoff to define the hotspots might look arbitrary. However, we considered that due the interindividual variability of PS values an absolute PS cutoff would not have been appropriate for the analysis. In addition, alternative cutoffs were explored in a prior phase to the analysis showing low sensitivity for more restrictive cutoffs (i.e., top 5 percentile PS values) and low specificity for more inclusive cutoffs (i.e., 15% percentile PS values). To improve the visual assessment and the noise produced by small areas of high PS, we applied a minimum volume threshold of 5 voxels. Third, the PS analysis was performed only in the white matter, but most of the SVD markers are represented in deep gray matter as well. However, as a first analysis of specific focal alteration of the BBB, we decided not to introduce additional variability in PS due to the different tissue characteristics between gray and white matter. Fourth, we were able to describe a colocalization between areas of high BBB leakage and SVD lesions. However, the cross-sectional design of the study precludes drawing causal relationships regarding the origin and progression of SVD lesions. For instance, future studies should assess the course of SVD lesions located in regions of high BBB leakage compared to others with more preserved BBB function. Fifthly, the subgroup analysis could not be performed according to SVD type due to the small sample size and the number of variables. To partially tackle this shortcoming, we provided 2 multivariable models differing in the inclusion of the SVD type variable.

To conclude, high PS values associate with worse SVD and the highest PS values may correspond to well-defined markers of SVD, such as lacunes, WMH, and WMH, independently of the aetiology of SVD. About half of patients with lacunes showed at least one of them surrounded by areas with highest PS values, which also seem to be associated with altered hemodynamics in the white matter characterized further by decreased cerebrovascular reactivity to vasoactive stimuli. About half of the patients showed hotspots in or at WMH edges, reinforcing the hypothesis that BBB impairment is a hallmark of white matter injury that may occur within WMH and in the contiguous NAWM. However, evidence from pathology studies showed no difference in tissular fibrinogen labelling, as a marker of BBB leakage between WMH and NAWM,^30^ suggesting that BBB impairment could be variable within both WMH and NAWM, and that regional spots of high BBB leakage could be more relevant in tissue injury than mean estimations from large areas. Nevertheless, the causative role of focal BBB impairment in the progression of SVD needs to be explored in longitudinal cohorts in the future.

## Supplemental Material

sj-pdf-1-jcb-10.1177_0271678X231173444 - Supplemental material for Blood-brain barrier leakage hotspots collocating with brain lesions due to sporadic and monogenic small vessel disease Click here for additional data file. Supplemental material, sj-pdf-1-jcb-10.1177_0271678X231173444 for Blood-brain barrier leakage hotspots collocating with brain lesions due to sporadic and monogenic small vessel disease by Salvatore Rudilosso, Michael S Stringer, Michael Thrippleton, Francesca Chappell, Gordon W Blair, Daniela Jaime Garcia, Fergus Doubal, Iona Hamilton, Esther Janssen, Anna Kopczak, Michael Ingrisch, Danielle Kerkhofs, Walter H Backes, Julie Staals, Marco Duering, Martin Dichgans, Joanna M Wardlaw and on behalf of the SVDs@target consortium in Journal of Cerebral Blood Flow & Metabolism
