# Supplementary material for: Blood-brain barrier leakage hotspots collocating with brain lesions due to sporadic and monogenic small vessel disease
Source: J Cereb Blood Flow Metab. 2023 May 3;43(9):1490–502. doi: 10.1177/0271678X231173444 (PMC10414006; doi:10.1177/0271678X231173444)
Supplement: sj-pdf-1-jcb-10.1177_0271678X231173444 - Supplemental material for Blood-brain barrier leakage hotspots collocating with brain lesions due to sporadic and monogenic small vessel disease [file sj-pdf-1-jcb-10.1177_0271678X231173444.pdf]

Online supplemental material

Hotspot load visual score:

For cerebellum and internal capsule hotspot load 4-level score was: absence, low (<4 clusters and no large confluent areas), medium (≥ 4 cluster or one large confluent area, but < 50% of the brain region), high (≥ 50% of the brain region or ≥ 2 large confluent areas), considering large confluent area a volume>200mm^3. For the rest of white matter regions: absence, low (<7 clusters and no large confluent areas), medium (≥ 7 cluster or 2 or 3 large confluent areas, but < 50% of the brain region), high (≥50% of the brain region or ≥ 3 large confluent areas), considering large confluent area a volume>400mm^3.

Table 1. Main clinical and radiological characteristics according to the lacune and WMH

	Patients included in the lacune analysis			Patients included in the WMH analysis		
	Sporadic SVD, N=24 (52.2%)	CADASIL, N=22 (47.8%)	p	Sporadic SVD, N= 40 (66.7%)	CADASIL, N= 20 (33.3%)	p
Age, y, mean (SD)	60.1 (10.4)	55.4 (8.7)	0.024	63.8 (10.9)	49.4 (10.6)	<0.001
Female, n (%)	8 (33.3)	9 (40.9)	0.595	18 (45.0)	10 (50.0)	0.714
Ischaemic heart disease, n (%)	4 (16.7)	1 (4.6)	0.349	7 (17.5)	1 (5.0)	0.249
Diabetes, n (%)	3 (12.5)	1 (4.5)	0.609	9 (22.5)	1 (5.0)	0.142
Hypertension, n (%)	19 (79.2)	9 (40.9)	0.008	33 (82.5)	7 (35.0)	<0.001
Hyperlipidaemia, n (%)	18 (75.0)	9 (40.9)	0.019	31 (77.5)	6 (30.0)	<0.001
Alcohol use, n (%)	18 (75.0)	17 (77.3)	1.857	27 (67.5)	14 (70.0)	0.844
Smoker, n (%)	13 (54.2)	11 (50.0)	0.595	19 (47.5)	9 (45.0)	0.855
Antiplatelet, n (%)	22 (91.7)	13 (59.1)	0.010	38 (95.0)	9 (45.0)	<0.001
Lacunes total, median (IQR)	3 (1-6)	7 (3-10)	0.023	1 (0-4)	4 (1-8.5)	0.064
PVH Fazekas score, median (IQR)	2 (1-2.5)	3 (3-3)	<0.001	2 (1-2)	3 (3-3)	<0.001
DWMH Fazekas score, median (IQR)	2 (1-2)	3 (3-3)	<0.001	1.5 (1-2)	3 (2-3)	<0.001
PVS in basal ganglia, median (IQR)	3 (1-3)	3 (2-4)	0.020	2 (1-2.5)	2 (2-3.5)	0.039
PVS in centrum semiovale, median (IQR)	3 (2-3)	3.5 (3-4)	0.015	2 (1-3)	3 (2-3.5)	0.051
CMB, median (IQR)	1 (0-5)	2.5 (0-9)	0.270	0 (0-2)	1 (0-3.5)	0.188
WMH volume, mL, median (IQR)	10.0 (5.0-15.3)	81.5 (49.1-147.4)	<0.001	8.5 (4.5-13.0)	53.7 (34.9-81.5)	<0.001

PS mean NAWM*10^4, mean (SD)	1.07 (0.93)	-0.19 (1.14)	<0.001	0.94 (0.96)	-0.25 (1.08)	<0.001
PS mean WMH*10^4, mean (SD)	1. 32 (1.01)	0.69 (0.89)	0.071	1.11 (1.22)	0.74 (0.91)	0.244
vP mean NAWM*10^4, mean (SD)	45.97 (12.72)	31.19 (10.87)	<0.001	47.12 (14.30)	34.49 (10.81)	0.001
vP mean WMH*10^4, mean (SD)	67.73 (21.04)	40.75 (15.97)	<0.001	71.45 (23.88)	53.51 (29.37)	0.017
CVR mean NAWM*10^2, mean (SD)	3.51 (3.98)	3.20 (3.81)	0.798	3.61 (3.24)	3.82 (3.10)	0.815
CVR mean WMH*10^2, mean (SD)	7.20 (6.47)	2.78 (6.60)	0.035	7.39 (6.30)	4.69 (6.04)	0.128
Number of hotspots, median (IQR)	57 (48-73)	82 (64-119)	<0.001	46.5 (46-68)	73.5 (59-89.5)	0.003

CADASIL: cerebral autosomal dominant arteriopathy with subcortical infarcts and leukoencephalopathy. WM: white matter; NAWM: normal appearing white matter; WMH: white matter hyperintensities; PVH: periventricular hyperintensities; DWHM: deep white matter hyperintensities; PVS: perivascular spaces; CMB: cerebral microbleeds; Normalized WMH: WMH volume / intracerebral volume; PS: permeability surface product; vP: vascular plasma volume; CVR: cerebrovascular reactivity, hPPS: high PS spots according to highest 10% PS values in segmented WM. CVR measures were calculated among the patients with full data available.

Table 2. Logistic regression for the presence of hotspots at lacune edges (n=46). Model 1 (adjusted for age, log 10 normalized WMH, number of lacunes, and CMB). Model 2 (adjusted for type of SVD, age, log 10 normalized WMH, number of lacunes, and CMB).

	Unadjusted OR	P value	Adjusted (model 1) OR	P value	Adjusted (model 2) OR	P value
Male	7.03 (1.84-26.85)	0.004	9.69 (2.06-45.49)	0.004	9.83 (2.08-46.46)	0.004
CADASIL	2.26 (0.66-7.76)	0.197	1.57 (0.07-34.12)	0.775	1.57 (0.07-34.12)	0.775
Hypertension	0.33 (0.09-1.25)	0.104	0.24 (0.05-1.21)	0.084	0.24 (0.05-1.21)	0.084
Number of lacunes	1.08 (0.95-1.21)	0.248	1.04 (0.90-1.20)	0.623	1.04 (0.90-1.20)	0.587
Log10 normWMH	1.46 (0.92-2.35)	0.112	1.65 (0.89-3.05)	0.109	1.43 (0.46-4.50)	0.539
PS mean NAWM*10^4	0.54 (0.29-1.00)	0.052	0.50 (0.21-1.19)	0.117	0.49 (0.20-1.19)	0.114
PS mean WMH*10^4	0.51 (0.24-1.08)	0.077	0.36 (0.12-1.10)	0.074	0.36 (0.12-1.19)	0.074
vP mean NAWM*10^4	0.99 (0.94-1.03)	0.572	1.00 (0.95-1.06)	0.861	1.00 (0.95-1.06)	0.811
vP mean WMH*10^4	0.98 (0.95-1.01)	0.142	0.99 (0.94-1.03)	0.509	0.99 (0.94-1.03)	0.504
CVR NAWM*10^2	0.67 (0.43-1.04)	0.075	0.64 (0.39-1.06)	0.082	0.64 (0.39-1.06)	0.083
CVR WMH*10^2	0.77 (0.63-0.94)	0.012	0.70 (0.53-0.93)	0.014	0.67 (0.50-0.92)	0.013

CADASIL: cerebral autosomal dominant arteriopathy with subcortical infarcts and leukoencephalopathy; NAWM: normal appearing white matter; WMH: white matter hyperintensities; CMB: cerebral microbleeds; PS: permeability surface product; vP: vascular plasma volume; CVR: cerebrovascular reactivity. CVR and were calculated among the patients with full data available.

Table 3. Ordinal regression for the number of hotspots at lacune edges (n=46). Model 1 (adjusted for age, log 10 normalized WMH, number of lacunes, and CMB). Model 2 (adjusted for type of SVD, age, log 10 normalized WMH, number of lacunes, and CMB).

	Unadjusted OR	P value	Adjusted (model 1) OR	P value	Adjusted (model 2) OR	P value
Male	6.50 (1.87- 22.57)	0.003	9.99 (2.44-40.87)	0.001	11.39 (2.68-48.29)	0.001
CADASIL	4.05 (1.31-12.52)	0.015	2.63 (0.33-21.13)	0.362	2.63 (0.33-21.13)	0.362
Hypertension	0.49 (0.17-1.46)	0.201	0.53 (0.14-1.94)	0.335	0.56 (0.15-2.07)	0.384
Number of lacunes	1.07 (0.99-1.16)	0.093	1.03 (0.94-1.13)	0.537	1.05 (0.95-1.18)	0.339
Log10 normWMH	1.68 (1.09-2.59)	0.018	1.57 (0.98-2.50)	0.059	1.15 (0.51-2.58)	0.741
PS mean NAWM*10^4	0.49 (0.28-0.84)	0.010	0.58 (0.29-1.17)	0.130	0.61 (0.30-1.27)	0.170
PS mean WMH*10^4	0.63 (0.36-1.11)	0.113	0.80 (0.38-1.69)	0.559	0.82 (0.39-1.74)	0.603
vP mean NAWM*10^4	0.99 (0.94-1.03)	0.572	1.00 (0.95-1.06)	0.861	1.01 (0.95-1.06)	0.811
vP mean WMH*10^4	0.98 (0.95-1.01)	0.142	0.99 (0.94-1.03)	0.509	0.99 (0.94-1.03)	0.504
CVR NAWM*10^2	0.89 (0.77-1.03)	0.130	0.89 (0.77-1.02)	0.103	0.89 (0.77-1.02)	0.105
CVR WMH*10^2	0.89 (0.81-0.97)	0.088	0.90 (0.81-0.99)	0.026	0.89 (0.81-0.98)	0.022

CADASIL: cerebral autosomal dominant arteriopathy with subcortical infarcts and leukoencephalopathy; NAWM: normal appearing white matter; WMH: white matter hyperintensities; CMB: cerebral microbleeds; PS: permeability surface product; vP: vascular plasma volume; CVR: cerebrovascular reactivity. CVR and were calculated among the patients with full data available.

Table 4. Logistic regression for the presence of hotspots within WMH (N=60). Model 1 (adjusted for age, log 10 normalized WMH, number of lacunes, and CMB). Model 2 (adjusted for type of SVD, age, log 10 normalized WMH, number of lacunes, and CMB).

	Unadjusted OR	P value	Adjusted (model 1) OR	P value	Adjusted (model 2) OR	P value
CADASIL	36 (6.89-188.13)	0.001	3.98 (0.24-65.70)	0.335	3.98 (0.24-65.70)	0.335
Age	0.95 (0.91.1.00)	0.040	0.99 (0.92-1.07)	0.827	0.98 (0.90-1.06)	0.619
Male sex	0.79 (0.28-2.20)	0.651	0.64 (0.12-3.34)	0.601	0.67 (0.13-3.55)	0.636
Number of lacunes	1.04 (0.95-1.14)	0.447	0.98 (0.80-1.20)	0.840	0.99 (0.89-1.22)	0.943
Log10 normWMH	5.10 (2.29-11.39)	<0.001	8.45 (2.68-26.68)	<0.001	5.74 (1.77-22.30)	0.012
Antiplatelets	0.25 (0.07-0.94)	0.041	4.66 (0.31-70.02)	0.266	+	
Hypertension	0.18 (0.06-0.59)	0.005	0.67 (0.11-3.97)	0.758	0.75 (0.12-4.67)	0.755
Hyperlipidaemia	0.39 (0.12-1.25)	0.112	0.07 (0.01-0.56)	0.012	0.07 (0.01-0.62)	0.016
PVS in basal ganglia, median (IQR)	1.69 (1.01-2.81)	0.44	1.40 (0.57-3.40)	0.460	1.34 (0.54-3.31)	0.525
PVS in centrum semiovale, median (IQR)	1.41 (0.84-2.39)	0.195	0.70 (0.29-1.70)	0.427	0.72 (0.29-1.78)	0.475
PS mean NAWM*10^4	0.49 (0.28-0.85)	0.012	0.70 (0.27-1.84)	0.471	0.79 (0.27-2.35)	0.673
PS mean WMH*10^4	0.91 (0.57-1.46)	0.700	1.52 (0.56-4.09)	0.410	1.61 (0.60-4.34)	0.342
vP mean NAMH*10^4	0.95 (0.90-0.99)	0.017	0.98 (0.92-1.04)	0.507	0.98 (0.92-1.05)	0.633
vP mean WMH*10^4	0.97 (0.94-0.99)	0.015	1.00 (0.96-1.05)	0.824	1.00 (0.96-1.05)	0.929
CVR NAWM*10^2	0.94 (0.78-1.12)	0.471	0.86 (0.68-1.08)	0.194	0.87 (0.69-1.10)	0.246
CVR WMH*10^2	0.92 (0.84-1.02)	0.112	0.97 (0.86-1.10)	0.682	0.97 (0.85-1.11)	0.692

CADASIL: cerebral autosomal dominant arteriopathy with subcortical infarcts and leukoencephalopathy; Normalized WMH: WMH volume / intracerebral volume PVS: perivascular spaces; NAWM: normal appearing white matter; WMH: white matter hyperintensities; CMB: cerebral microbleeds; PS: permeability surface product; vP: vascular plasma volume; CVR: cerebrovascular reactivity. CVR and were calculated among the patients with full data available. + Not estimable for data quasi separation

Table 5. Ordinal regression for the number of hotspots within WMH (N=60). Model 1 (adjusted for age, log 10 normalized WMH, number of lacunes, and CMB). Model 2 (adjusted for type of SVD, age, log 10 normalized WMH, number of lacunes, and CMB).

	Unadjusted OR	P value	Adjusted (model 1) OR	P value	Adjusted (model 2) OR	P value
CADASIL	60.05 (12.48-288.87)	<0.001	3.75 (0.33-42.03)	0.283	3.75 (0.33-42.03)	0.283
Age	0.94 (0.90-0.99)	0.009	0.94 (0.90-0.99)	0.024	0.97 (0.90-1.03)	0.320
Male sex	0.78 (0.29-2.09)	0.620	0.67 (0.18-2.48)	0.552	0.64 (0.17-2.35)	0.500
Number of lacunes	1.03 (0.94-1.13)	0.533	0.86 (0.72-1.03)	0.099	0.88 (0.74-1.05)	0.145
Log10 normWMH	5.52 (2.77-11.02)	<0.001	9.12 (3.66-22.75)	<0.001	6.28 (2.06-19.12)	0.001
Antiplatelets	0.26 (0.08-0.81)	0.021	2.75 (0.57-13.33)	0.210	4.52 (0.78-26.30)	0.094
Hypertension	0.18 (0.06-0.53)	0.002	1.32 (0.31-5.55)	0.704	1.54 (0.36-6.65)	0.560
Hyperlipidaemia	0.15 (0.05-0.44)	0.001	0.42 (0.10-1.73)	0.231	0.43 (0.10-1.76)	0.239
PVS in basal ganglia, median (IQR)	1.66 (1.02-2.70)	0.040	1.51 (0.75-3.04)	0.245	1.42 (0.71-2.87)	0.325
PVS in centrum semiovale, median (IQR)	1.49 (0.88-2.51)	0.134	0.71 (0.36-1.40)	0.319	0.71 (0.35-1.41)	0.324
PS mean NAWM*104	0.35 (0.19-0.62)	<0.001	0.36 (0.16-0.81)	0.014	0.35 (0.14-0.86)	0.022
PS mean WMH*10^4	0.77 (0.48-1.24)	0.287	0.79 (0.39-1.59)	0.508	0.81 (0.40-1.61)	0.545
vP mean NAMH*10^4	0.95 (0.90-0.99)	0.019	1.01 (0.96-1.06)	0.795	1.02 (0.96-1.07)	0.598
vP mean WMH*10^4	0.97 (0.94-0.99)	0.010	1.04 (0.99-1.08)	0.109	1.03 (0.99-1.08)	0.133
CVR NAWM*10^2	1.01 (0.87-1.19)	0.871	0.96 (0.79-1.17)	0.676	0.95 (0.78-1.16)	0.638
CVR WMH*10^2	0.94 (0.87-1.03)	0.192	1.02 (0.90-1.15)	0.804	1.00 (0.88-1.14)	0.994

CADASIL: cerebral autosomal dominant arteriopathy with subcortical infarcts and leukoencephalopathy; Normalized WMH: WMH volume / intracerebral volume PVS: perivascular spaces; NAWM: normal appearing white matter; WMH: white matter hyperintensities; CMB: cerebral microbleeds; PS: permeability surface product; vP: vascular plasma volume; CVR: cerebrovascular reactivity. CVR and were calculated among the patients with full data available. + Not estimable for data quasi separation

Table 6. Logistic regression for the presence of hotspots at WMH edges (N=60). Model 1 (adjusted for age, log 10 normalized WMH, number of lacunes, and CMB). Model 2 (adjusted for type of SVD, age, log 10 normalized WMH, number of lacunes, and CMB).

	Unadjusted OR	P value	Adjusted (model 1) OR	P value	Adjusted (model 2) OR	P value
CADASIL	4.89 (1.39-17.24)	0.014	1.40 (0.13-15.00)	0.781	1.40 (0.13-15.00)	0.781
Age	1.00 (0.96-1.04)	0.998	1.02 (0.97-1.06)	0.493	1.02 (0.96-1.09)	0.482
Male sex	1.27 (0.45-3.53)	0.651	1.10 (0.31-3.88)	0.882	1.11 (0.31-3.92)	0.872
Number of lacunes	1.07 (0.96-1.20)	0.240	0.96 (0.85-1.08)	0.487	0.96 (0.85-1.09)	0.552
Log10 normWMH	2.79 (1.55-5.02)	0.001	3.13 (1.61-6.06)	0.001	2.87 (1.19-6.95)	0.019
Antiplatelets	0.51 (0.13-1.87)	0.307	0.89 (0.13-6.29)	0.906	0.97 (0.12-7.81)	0.976
Hypertension	0.20 (0.06-0.72)	0.014	0.22 (0.04-1.16)	0.075	0.22 (0.04-1.19)	0.079
Hyperlipidaemia	0.56 (0.19-1.65)	0.294	0.90 (0.19-4.19)	0.896	0.93 (0.20-4.44)	0.931
PVS in basal ganglia, median (IQR)	1.42 (0.86-2.36)	0.174	0.83 (0.39-1.74)	0.614	0.81 (0.38-1.72)	0.588
PVS in centrum semiovale, median (IQR)	1.63 (0.95-2.80)	0.075	1.11 (0.55-2.51)	0.767	1.12 (0.55-2.62)	0.757
PS mean NAWM*10^4	1.28 (0.80-2.06)	0.297	4.03 (1.53-10.57)	0.005	5.13 (1.69-15.56)	0.004
PS mean WMH*10^4	1.52 (0.91-2.52)	0.108	3.79 (1.38-10.43)	0.010	4.14 (1.42-12.06)	0.009
vP mean NAMH*10^4	0.99 (0.96-1.03)	0.765	1.04 (0.99-1.10)	0.133	1.04 (0.99-1.10)	0.134
vP mean WMH*10^4	0.98 (0.96-1.00)	0.043	1.01 (0.98-1.04)	0.526	1.01 (0.98-1.04)	0.533
CVR WM*10^2	1.05 (0.88-1.25)	0.567	1.10 (0.90-1.35)	0.355	1.11 (0.89-1.37)	0.353
CVR WMH*10^2	1.01 (0.93-1.11)	0.760	1.12 (1.00-1.27)	0.051	1.13 (1.00-1.27)	0.052

CADASIL: cerebral autosomal dominant arteriopathy with subcortical infarcts and leukoencephalopathy; Normalized WMH: WMH volume / intracerebral volume PVS: perivascular spaces; NAWM: normal appearing white matter; WMH: white matter hyperintensities; CMB: cerebral microbleeds; PS: permeability surface product; vP: vascular plasma volume; CVR: cerebrovascular reactivity. CVR and were calculated among the patients with full data available. + Not estimable for data quasi separation

Table 7. Ordinal regression for the number of of hotspots at WMH edges (N=60). Model 1 (adjusted for age, log 10 normalized WMH, number of lacunes, and CMB). Model 2 (adjusted for type of SVD, age, log 10 normalized WMH, number of lacunes, and CMB).

	Unadjusted OR	P value	Adjusted (model 1) OR	P value	Adjusted (model 2) OR	P value
CADASIL	5.59 (1.97-15.85)	0.001	1.79 (0.27-11.96)	0.547	1.79 (0.27-11.96)	0.547
Age	0.99 (0.96-1.03)	0.778	1.01 (0.97-1.05)	0.509	1.02 (0.97-1.08)	0.373
Male sex	1.29 (0.52-3.24)	0.581	0.88 (0.32-2.48)	0.814	0.87 (0.31-2.44)	0.791
Number of lacunes	1.09 (1.00-1.17)	0.041	1.01 (0.97-1.05)	0.509	0.97 (0.87-1.08)	0.373
Log10 normWMH	3.12 (1.89-5.15)	<0.001	3.53 (1.98-6.28)	<0.001	2.99 (1.38-6.47)	0.005
Antiplatelets	0.43 (0.14-1.31)	0.139	0.74 (0.17-3.19)	0.688	0.84 (0.18-3.88)	0.820
Hypertension	0.23 (0.09-0.64)	0.004	1.02 (0.98-1.07)	0.239	1.03 (0.98-1.08)	0.273
Hyperlipidaemia	0.46 (0.18-1.20)	0.112	0.64 (0.19-2.10)	0.462	1.03 (0.98-1.09)	0.284
PVS in basal ganglia, median (IQR)	1.60 (1.00-2.57)	0.050	0.91 (0.50-1.65)	0.756	0.89 (0.48-1.63)	0.701
PVS in centrum semiovale, median (IQR)	1.62 (1.00-2.64)	0.052	0.98 (0.55-1.75)	0.942	0.98 (0.55-1.75)	0.946
PS mean NAWM*10^4	1.05 (0.68-1.63)	0.819	1.78 (0.99-3.23)	0.056	1.91 (1.04-3.52)	0.038
PS mean WMH*10^4	1.29 (0.82-2.05)	0.272	1.81 (0.99-3.30)	0.054	1.85 (1.01-3.39)	0.047
vP mean NAMH*10^4	0.99 (0.96-1.03)	0.722	1.04 (0.99-1.08)	0.080	1.04 (0.99-1.08)	0.058
vP mean WMH*10^4	0.98 (0.96-0.99)	0.021	1.01 (0.98-1.04)	0.386	1.01 (0.98-1.04)	0.409
CVR WM*10^2	1.03 (0.88-1.21)	0.722	1.11 (0.93-1.32)	0.251	1.12 (0.94-1.34)	0.213
CVR WMH*10^2	0.98 (0.91-1.06)	0.595	1.09 (0.99-1.20)	0.073	1.09 (0.99-1.19)	0.063

CADASIL: cerebral autosomal dominant arteriopathy with subcortical infarcts and leukoencephalopathy; Normalized WMH: WMH volume / intracerebral volume PVS: perivascular spaces; NAWM: normal appearing white matter; WMH: white matter hyperintensities; CMB: cerebral microbleeds; PS: permeability surface product; vP: vascular plasma volume; CVR: cerebrovascular reactivity. CVR and were calculated among the patients with full data available. + Not estimable for data quasi separation

Table 8. The SVDs@target Investigators

Name and Degree	Affiliation	Role	Contribution
Martin Dichgans, MD	Institute for Stroke and Dementia Research (ISD), University Hospital, LMU Munich, 81377 Munich, Germany.	PI	Funding, regulatory, protocol, oversight
Anna Kopczak, MD	Institute for Stroke and Dementia Research (ISD), University Hospital, LMU Munich, 81377 Munich, Germany.	Investigator	Protocol, patient recruitment, assessment, analysis
Marco Düring, MD	Institute for Stroke and Dementia Research (ISD), University Hospital, LMU Munich, 81377 Munich, Germany.	Investigator	scanning
Benno Gesierich, PhD	Institute for Stroke and Dementia Research (ISD), University Hospital, LMU Munich, 81377 Munich, Germany.	Investigator	scanning
Karin Waegemann, PhD	Institute for Stroke and Dementia Research (ISD), University Hospital, LMU Munich, 81377 Munich, Germany.	Investigator	Study management,
Michael Ingrisch, PhD	Department of Clinical Radiology, LMU-University of Munich, 81377 Munich, Germany	Investigator	scanning
Joanna M Wardlaw, MD	Centre for Clinical Brain Sciences, University of Edinburgh, Edinburgh, UK; UK DRI at the University of Edinburgh, University of Edinburgh, Edinburgh, UK.	PI	Funding, regulatory, protocol, analysis, oversight
Gordon W Blair, MD	Centre for Clinical Brain Sciences, University of Edinburgh, Edinburgh, UK; UK DRI at the University of Edinburgh, University of Edinburgh, Edinburgh, UK.	Investigator	Protocol, patient recruitment, assessment, analysis

Fergus N Doubal MD	Centre for Clinical Brain Sciences, University of Edinburgh, Edinburgh, UK; UK DRI at the University of Edinburgh, University of Edinburgh, Edinburgh, UK.	Investigator	Protocol, patient oversight,
Michael S Stringer, PhD	Centre for Clinical Brain Sciences, University of Edinburgh, Edinburgh, UK; UK DRI at the University of Edinburgh, University of Edinburgh, Edinburgh, UK.	Investigator	scanning
Michael J Thrippleton, PhD	Centre for Clinical Brain Sciences, University of Edinburgh, Edinburgh, UK; UK DRI at the University of Edinburgh, University of Edinburgh, Edinburgh, UK.	Investigator	scanning
Francesca Chappell, PhD	Centre for Clinical Brain Sciences, University of Edinburgh, Edinburgh, UK; UK DRI at the University of Edinburgh, University of Edinburgh, Edinburgh, UK.	Investigator	Analysis, data management
Iona F Hamilton, DCR	Centre for Clinical Brain Sciences, University of Edinburgh, Edinburgh, UK; UK DRI at the University of Edinburgh, University of Edinburgh, Edinburgh, UK.	Investigator	scanning
Dany Jaime Garcia, MSc	Centre for Clinical Brain Sciences, University of Edinburgh, Edinburgh, UK; UK DRI at the University of Edinburgh, University of Edinburgh, Edinburgh, UK.	Investigator	patient recruitment, assessment, analysis
Carmen Arteaga Reyes, MB ChB	Centre for Clinical Brain Sciences, University of Edinburgh, Edinburgh, UK; UK DRI at the University of Edinburgh, University of Edinburgh, Edinburgh, UK.	Investigator	patient recruitment, assessment, analysis
Salvatore Rudilosso, PhD	Comprehensive Stroke Centre, Hospital Clinic, University of Barcelona, Spain	Investigator	analysis
Ernest Chui, BSc	Medical School, University of Edinburgh, Edinburgh	Student	analysis
Robert J van Oostenbrugge, MD	Department of Neurology, Maastricht University Medical Center, the Netherlands	PI	Funding, regulatory, protocol, oversight
Julie Staals, MD	Department of Neurology, Maastricht University Medical Center, the Netherlands	Investigator	Protocol, patient oversight

Maud van Dinther, MSc	Department of Neurology, Maastricht University Medical Center, the Netherlands	Investigator	analysis
Danielle Kerkhofs, MB ChB	Department of Neurology, Maastricht University Medical Center, the Netherlands	Investigator	Protocol, patient recruitment, assessment, analysis
Walter H Backes, PhD	Department of Medical Physics, Maastricht University Medical Center, Maastricht, The Netherlands	Investigator	scanning
Geert Jan Biessels, MD	Department of Neurology, UMC Utrecht Brain Center, University Medical Center Utrecht, the Netherlands	PI	Funding, regulatory, protocol,
Hilde van den Brink, MSc	Department of Neurology and Neurosurgery, UMC Utrecht Brain Center, University Medical Center Utrecht, Utrecht, The Netherlands.	Investigator	Patient recruitment, assessment, analysis
Laurien P Onkenhout, MSc	Department of Neurology and Neurosurgery, UMC Utrecht Brain Center, University Medical Center Utrecht, Utrecht, The Netherlands.	Investigator	analysis
Tine Arts, PhD	Department of Imaging Sciences, University Medical Center Utrecht, Utrecht, The Netherlands	Investigator	Scanning, analysis
Stanley DT Pham, PhD	Department of Imaging Sciences, University Medical Center Utrecht, Utrecht, The Netherlands	Investigator	analysis
Jeroen Hendrikse, MD	Department of Imaging Sciences, University Medical Center Utrecht, Utrecht, The Netherlands	Investigator	Protocol, analysis
Jaco JMZ Zwanenburg, PhD	Department of Imaging Sciences, University Medical Center Utrecht, Utrecht, The Netherlands	Investigator	Scanning, analysis
Jeroen CW Siero, PhD	Department of Imaging Sciences, University Medical Center Utrecht, Utrecht, The Netherlands	Investigator	analysis
Alastair JS Webb, MD	Department of Clinical Neurosciences, Wolfson Centre for Prevention of Stroke and Dementia, University of Oxford, UK	PI	Protocol, analysis
